# Effect of race and sex on lupus diagnosis in primary care: A randomized factorial survey study

**DOI:** 10.1371/journal.pone.0342328

**Published:** 2026-02-06

**Authors:** Alyssa Howren, Quan L. Tran, Sadaf Sediqi, Saadiya Hawa, Douglas K. Owens, Eleni Linos, Titilola O. Falasinnu, Yashaar Chaichian, Julia F. Simard

**Affiliations:** 1 Department of Epidemiology and Population Health, Stanford School of Medicine, Stanford, California, United States of America; 2 Department of Medicine, Stanford School of Medicine, Stanford, California, United States of America; 3 Graduate Medical Education, Department of Internal Medicine, Weiss Memorial Hospital, Chicago, Illinois, United States of America; 4 Department of Health Policy, Stanford School of Medicine, Stanford, California, United States of America; 5 Department of Dermatology, Stanford School of Medicine, Stanford, California, United States of America; 6 Department of Anesthesiology, Perioperative and Pain Medicine, Stanford School of Medicine, Stanford, California, United States of America; 7 Division of Immunology and Rheumatology, Department of Medicine, Stanford School of Medicine, Stanford, California, United States of America; Prime Hospital LLC, UNITED ARAB EMIRATES

## Abstract

**Background:**

Systemic lupus erythematosus (SLE) is a heterogeneous autoimmune rheumatic disease whose epidemiology and clinical prognosis vary by race and sex. Observed disparities in SLE may be partly attributable to cognitive processes in clinical decision-making, which can influence diagnostic accuracy and clinical management. We aimed to examine variation in primary care physicians’ (PCP) diagnosis and management of SLE when all content of a clinical case is identical, apart from race and sex.

**Methods:**

We distributed an online randomized factorial survey from 04/11/2024–06/10/2024 to PCPs across the US. Participants were presented with one of four possible SLE vignettes – Black female, White female, Black male, White male – for which all other clinical content was identical. Block randomization was used to randomly modify the race (Black/White) and sex (female/male) of the SLE “case”. Primary outcomes were correct text-based responses for SLE diagnosis at initial case presentation and after reviewing additional lab results. Secondary outcomes were participants’ review time and planned next steps (treatment, referral, tests) as a proxy for cognitive bias and certainty, respectively. We calculated descriptive statistics for all outcomes stratified by assigned randomized factor and used chi-square tests to evaluate between-group differences.

**Results:**

1031 PCPs (42.7% women, mean age 52.1 ± 12.1 years) completed the case. At initial presentation, 63.9% of participants correctly identified SLE as a differential diagnosis. An initial diagnosis of SLE significantly differed by the race and sex of the case (p < 0.001), with the highest proportion of correct diagnoses occurring for Black female cases (72.2%) and lowest for White male cases (55.3%). Median review time for correct initial diagnoses was longest for White male cases (175 s). After participants reviewed lab results, the overall proportion assigning a final diagnosis of SLE (63.9%) remained unchanged from the initial diagnosis.

**Conclusion:**

A patient’s race and sex may influence diagnostic accuracy and clinical decision-making for SLE in primary care. The observed variation in diagnostic accuracy, which aligns with the descriptive epidemiology of SLE, highlights the need for targeted interventions to ensure equitable diagnostic processes.

## Introduction

Systemic lupus erythematosus (SLE) is a heterogeneous autoimmune rheumatic disease whose epidemiology and clinical prognosis vary by race and sex. SLE is a female-predominant disease, with CDC National Lupus Registries estimating a nine-fold higher prevalence for females than males (128.7 versus 14.6 per 100,000) [1]. The California Lupus Surveillance Project reported a six-fold higher incidence rate among Black females compared to White females (30.5 versus 5.3 per 100,000 person-years) [2]. These estimates far exceed incidence of SLE among Black (2.1 per 100,000 person-years) and White males (0.6 per 100,000 person-years) [2]. Nonetheless, males are more likely to have severe manifestations at diagnosis, such as lupus nephritis and thrombocytopenia, and have a poorer prognosis [1,3,4].

Biological factors and social determinants are thought to underlie sex- and gender-based disparities in SLE epidemiology and clinical outcomes [5]. Adherence to traditional masculine norms and decreased health-seeking behaviors may also contribute to greater disease severity and poorer prognosis at the time of a clinical diagnosis in males [6,7]. Further, racial differences in SLE severity have been attributed to social determinants of health, including dimensions of socioeconomic status and racial discrimination [8]. Additionally these disparities, to some degree, may arise from cognitive errors during clinical decision-making [9,10]. Is it possible that physicians place selective attention on the “classic” case of SLE, thereby delaying diagnosis among less representative cases and inadvertently perpetuating these disparities? This representativeness bias was observed in pilot work showing rheumatologists were less likely to diagnose SLE for hypothetical male cases, particularly White males, despite identical clinical history, symptoms, and serology [10]. To expand on this work, we conducted a factorial survey experiment to examine whether randomly assigned patient race and sex affect the likelihood that primary care physicians (PCPs), who are often the first point of care, accurately diagnose SLE when all other clinical content remains identical. Secondarily, we investigated how PCPs subsequent actions, including ordering tests and referrals, varied by the randomly assigned race and sex of the case.

## Materials and methods

### Survey design

We developed an online factorial survey experiment using Qualtrics (Qualtrics, Provo, UT) comprising six clinical vignettes, one of which described SLE. We modified an existing SLE vignette from our rheumatologist experiment to better represent a primary care setting [10]. Principal clinical features of SLE described in the vignette included history of fever, intermittent joint pain and stiffness, photosensitivity but no rash, and fatigue (**S1 Appendix**). For each survey participant, the race (Black/White) and sex (female/male) of the “case” were randomly assigned, and all other social and clinical content describing symptoms, medical history, physical exam, and laboratory results remained constant. Participants were first presented with symptoms, medical history, and physical exam findings and asked, “What are the top three diagnoses on your differential?” and then, “What do you plan to do next?” with options to either observe and see how the disease progresses or conduct further investigations. Those electing further investigation were asked to choose and elaborate on immediate next step(s): order testing, initiate treatment, and/or refer. Finally, participants were presented additional laboratory findings and asked, “What is the most likely diagnosis?” (**S1 Fig**).

The survey was pilot tested among 13 PCPs affiliated with Stanford Medicine blinded to the survey’s purpose from 02/12/2024–03/13/2024 to assess the block randomization and optimize the clinical vignettes. Pilot stage participants received a $20 incentive, and their survey data were excluded from the analysis.

### Survey distribution

The survey was emailed by the research team through Qualtrics between 04/11/2024 and 05/22/2024 to US PCPs using an email distribution list from a medical marketing company (Lake B2B/Span Global Services). Participants were initially offered a $30 incentive distributed by Tango Rewards, which was increased to $50 on 05/06/2024 to help reach a target of 1000 respondents. Altogether, five reminder emails were sent, and the survey closed 06/10/2024.

### Study population

Eligible participants were those identifying as a PCP (family medicine/general practice, or internal medicine residency) at initial screening. We excluded participants living outside the US (n = 4), giving insincere responses (e.g., numbers, ‘X’, cancer for all diagnoses, n = 2), and reporting an improbable year of birth (e.g., 1900, n = 1) (**S2 Fig**).

### Measurements

#### Participant demographics.

Participants first answered questions on personal demographics, including age, gender, race, ethnicity, and residence. Information on participants’ clinical experience and practice were also collected, namely: years since completing residency; board certification; where their medical degree was obtained; practice location; practice setting; and focus of work.

#### Exposure.

We used Qualtrics’ block randomization to randomly assign the race (Black/White) and sex (female/male) of each case vignette shown to participants. Participants were presented with one of four possible SLE vignettes – Black female, White female, Black male, White male – for which all other content was identical.

#### Study outcomes.

The primary outcomes were correct text-based responses for SLE diagnosis after initial case presentation (i.e., top three differential diagnoses) and after reviewing additional lab results (i.e., final diagnosis). The classification of correct and incorrect responses was determined independently from the randomly assigned sex and race of the case. One author (AH) completed initial categorization, and all diagnostic categories were reviewed by three co-authors (AH, YC, JFS). For correct initial diagnoses, we derived a binary variable to indicate whether a diagnosis of SLE was listed in the top three differential, regardless of assigned rank. We subsequently followed a deductive approach to categorize incorrect responses into several binary disease categories (**S2 Appendix**).

We measured participants’ review time and planned next steps as a proxy for cognitive bias and certainty, respectively. Participants’ review time for each survey question was automatically measured in seconds. Participants’ next steps were first assessed by deriving non-mutually exclusive binary variables to indicate whether participants elected to order tests, send referral(s), and/or initiate treatment(s). We then classified participants detailed entries for next steps as binary variables. For example, derived variables indicated whether tests for antinuclear antibodies (ANA) were ordered, or a treatment, such as non-steroidal anti-inflammatory drugs (NSAIDs) was initiated (**S2 Appendix**).

We collectively captured final diagnoses with any mention of SLE. We then distinguished between responses that singularly entered SLE (e.g., “lupus”) from those including SLE with another differential (e.g., “SLE, seronegative RA”). Incorrect responses were assigned to binary disease categories (see **S2 Appendix**).

### Statistical analysis

Participant data were exported from Qualtrics into SAS version 9.4 (SAS Institute Inc., Cary, NC) for data preparation and analyses. Participant demographics were summarized with descriptive statistics. We calculated descriptive statistics for all study outcomes stratified by the randomly assigned race-sex version. We assessed for heterogeneity with respect to the proportion of correct initial and final diagnoses by race-sex version using chi-square tests. Given the initial clinical context of the vignette, which included joint pain and photosensitivity, the anticipated next step endorsed by Siegel et al.’s [11] SLE clinical review is for PCPs to check ANA level. We therefore evaluated the proportion of participants checking ANA level stratified by correct initial diagnosis and race-sex version of the case.

### Ethical approval and participant consent

This study was approved by the Institutional Review Board of Stanford University, Stanford, California (IRB – 42909). Participants completed the written informed consent process online prior to beginning the survey.

## Results

In total, 1031 PCPs (42.7% women, 52.1 ± 12.1 years) finished the survey. Most completed residency ≥11 years ago (76.4%) and indicated patient care was their main focus (85.7%) (**Table 1** and **S1 Table** for stratification by race-sex case version).

**Table 1:** Participant demographics (n = 1031).

Characteristic	
Age, mean (SD)	52.1 (12.1)
**Gender, No. (%)**	
Woman	440 (42.7)
Man	588 (57.0)
Not listed	3 (0.3)
**Race, No. (%)**	
White	700 (67.9)
Asian	252 (24.4)
Black	25 (2.4)
American Indian or Alaska Native	2 (0.2)
Pacific Islander or Native Hawaiian	2 (0.2)
≥2 selected	17 (1.6)
Not listed	33 (3.2)
**Ethnicity, No. (%)**	
Hispanic	50 (4.9)
Not Hispanic	981 (95.2)
**Medical school, No. (%)**	
US	764 (74.1)
International	267 (25.9)
**Years since residency, No. (%)**	
Currently in residency	28 (2.7)
<5 years	80 (7.8)
5-10 years	135 (13.1)
11-20 years	298 (28.9)
>20 years	490 (47.5)
**Practice setting, No. (%)**	
Private group practice	373 (36.2)
Academic	317 (30.8)
Multiple settings	125 (12.1)
City or County public hospital	85 (8.2)
Retired	42 (4.1)
Veteran Affairs	38 (3.7)
HMO	32 (3.1)
Military	1 (0.1)
Not working (looking to work)	4 (0.4)
Not working	4 (0.4)
Not clinically practicing	10 (1.0)
**Clinical work, No. (%)**	
Patient care	883 (85.7)
Administrative work	62 (6.0)
Education	60 (5.8)
Research	26 (2.5)
**Location of current practice, No. (%)**	
Urban	458 (44.4)
Suburban	409 (39.7)
Rural	164 (15.9)
**US geographic division, No. (%)**	
South Atlantic	182 (17.7)
Pacific	179 (17.4)
Middle Atlantic	147 (14.3)
East North Central	138 (13.4)
West North Central	123 (11.9)
Mountain	94 (9.1)
West South Central	71 (6.9)
New England	64 (6.2)
East South Central	33 (3.2)
Puerto Rico	0 (0.0)

Abbreviations: SD – standard deviation

At initial case presentation, 63.9% (659/1031) of participants correctly identified SLE within their top three differentials. SLE identification at this stage significantly differed by the race and sex of the case, with the highest proportion of correct diagnoses occurring for the Black female version (**Table 2**). Participants were more likely to rank SLE as the first differential diagnosis for Black female cases (31.6%), while SLE tended to rank as the second differential diagnosis for the White female (33.6%), Black male (24.9%), and White male cases (28.2%) (**Table 2**). Median review time among participants with correct initial diagnoses was the longest for White male vignettes (**Table 3**).

**Table 2:** Proportion of correct diagnoses identified among SLE clinical vignettes with race and sex jointly randomized.

	Race and Sex			
Measure	Black female	White female	Black male	White male
**Initial Diagnosis**				
Randomized, No.	263	253	253	262
Correct diagnosis within differential, No. (%)	190 (72.2)	172 (68.0)	152 (60.1)	145 (55.3)
*P* for heterogeneity	<0.001			
Correct 1^st^ diagnosis, No. (%)	83 (31.6)	57 (22.5)	55 (21.7)	28 (10.7)
Correct 2^nd^ diagnosis, No. (%)	81 (30.8)	85 (33.6)	63 (24.9)	74 (28.2)
Correct 3^rd^ diagnosis, No. (%)	26 (9.9)	30 (11.9)	34 (13.4)	43 (16.4)
**Final Diagnosis**				
Randomized, No.	263	253	253	262
Diagnosis of SLE (+/- differential)^a^, No. (%)	183 (69.6)	167 (66.0)	153 (60.5)	156 (59.5)
*P* for heterogeneity	0.055			
Diagnosis of SLE-specific^b^, No. (%)	161 (61.2)	144 (56.9)	133 (52.6)	129 (49.2)
*P* for heterogeneity	0.035			

^a^ *SLE (+/- differential) refers to participant responses that included SLE and may have also included an additional differential diagnosis (e.g., viral illness).*

^b^ *SLE-specific refers to participant responses that definitively stated SLE.*

Abbreviations: SLE – systemic lupus erythematosus

**Table 3:** Median time (in seconds) for participants to establish an initial and a final diagnosis.

	Race and Sex			
Measure	Black female	White female	Black male	White male
**Initial Diagnosis**				
Correct^a^, seconds (IQR)	154.0 (97.7-277.9)	133.0 (86.1-224.7)	139.4 (87.9-219.9)	175.2 (117.2-348.2)
Incorrect, seconds (IQR)	175.4 (100.5-261.5)	152.5 (82.7-242.7)	138.1 (96.3-256.7)	167.5 (117.2-267.6)
**Final Diagnosis**				
Correct^a^, seconds (IQR)	49.4 (32.7-94.7)	48.6 (30.9-71.0)	48.7 (29.5-88.5)	55.1 (34.5-115.8)
Incorrect, seconds (IQR)	59.3 (40.2-114.4)	54.4 (37.5-86.5)	65.6 (35.9-104.2)	65.9 (40.7-129.5)

^a^ *Correct if SLE included in top three initial differential diagnosis independent of assigned rank.*

^b^ *Correct if response listed SLE and may have also included an additional differential diagnosis.*

Abbreviations: IQR – interquartile range; SLE – systemic lupus erythematosus

Most participants included one or more other rheumatic diseases in their initial differential diagnosis (92.2%, 951/1031) (**S2 Table**). When SLE was not an initial differential, 87.9% (327/372) listed another rheumatic disease. Conditions less related to SLE, such as sexually transmitted infections, were included as a differential diagnosis disproportionately (15.8% Black male, 11.8% White male, 8.8% Black female, and 5.1% White female cases). Sarcoidosis was a differential diagnosis for a small margin of participants (21/1031, 2.0%), predominately for Black male (9/21, 42.9%) and Black female cases (10/21, 47.6%).

Upon initial case presentation, most participants elected to order tests (91.9%, 947/1031) and only 7.9% (81/1031) chose to send a referral (95.1% were to rheumatology (77/81)). Overall, 69.5% (717/1031) of participants ordered an ANA test. Among participants with SLE as an initial differential diagnosis, 78.9% (520/659) listed ANA in their lab orders and 9.0% (59/659) ordered rheumatology panels. Participants including SLE on their differential more frequently ordered ANA tests for White male cases (84.8%, 123/145), followed by Black female (80.0%, 152/190), Black male (75.7%, 115/152), and White female (75.6%, 130/172) cases. Fewer PCPs ordered ANA tests (53.0%, 197/372) when SLE was not a differential. A small proportion of participants (5.6%, 58/1031) elected to initiate treatment, most commonly NSAIDs (51.7%, 30/58) and corticosteroids (24.1%, 14/58).

**Fig 1:**
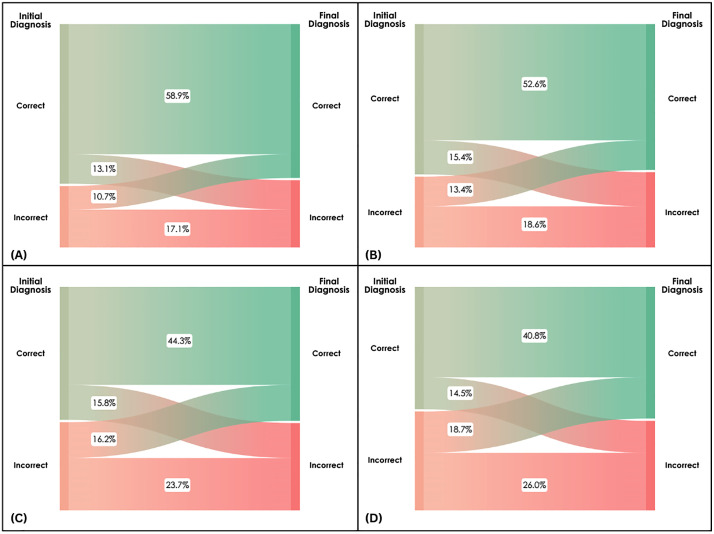
Flow of participant correct and incorrect responses from initial to final diagnosis presented according to the jointly randomized race and sex version of the SLE case vignette. **(A)** Black female case, **(B)** White female case, **(C)** Black male case, and **(D)** White male case.

**Fig 2:**
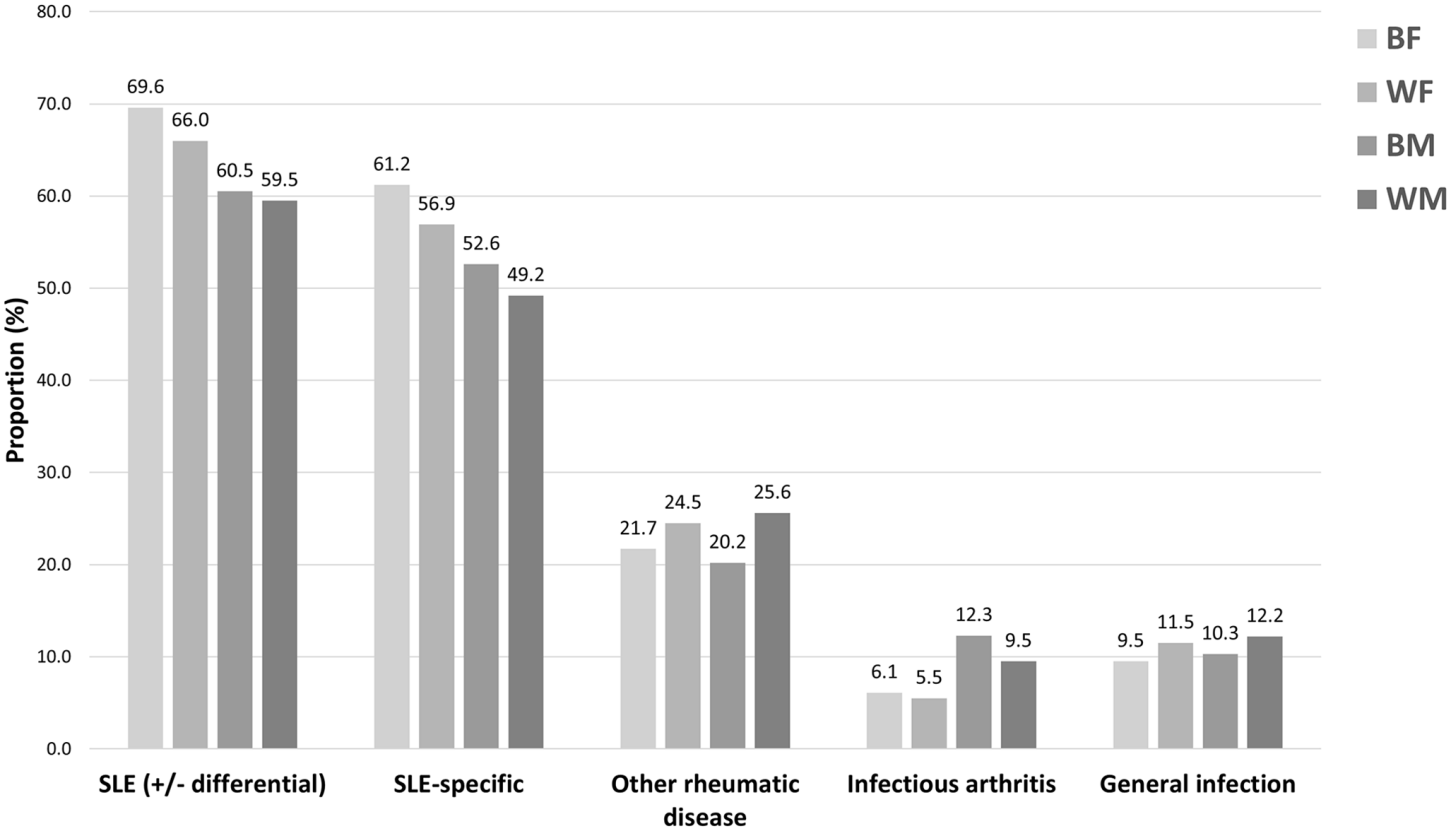
Participants’ final diagnosis for SLE clinical vignettes with race and sex jointly randomized. Presented diagnoses are for categories that were recorded by ≥5% of participants. Responses for ‘other rheumatic disease’ included rheumatoid arthritis, osteoarthritis, inflammatory arthritis, Behcet’s, and connective tissue diseases. All diagnostic categories aside from SLE-specific are not mutually exclusive. SLE (+/- differential) refers to participant responses that included SLE and may have also included an additional differential diagnosis. SLE-specific refers to participant responses that definitively stated SLE. Abbreviations: BF – Black Female; BM – Black Male; SLE – systemic lupus erythematosus; WF – White Female; WM – White Male.

After reviewing lab results, the net final diagnosis of SLE (63.9%, 659/1031) was similar to the initial diagnosis. This was not entirely due to participants maintaining their initial answers. A subset of PCPs switched between correct and incorrect diagnoses (**Fig 1**). Overall, a final diagnosis of SLE occurred more often for female versus male vignettes (**Table 2**). Other rheumatic diseases (e.g., rheumatoid arthritis) were the final diagnoses assigned by 23.0% (237/1031) of participants. Infectious arthritis and general infections were common incorrect diagnoses for Black and White male cases, respectively (**Fig 2**). The time to correct diagnosis in the final stage was again longest for White male vignettes (**Table 3**).

## Discussion

Despite identical clinical information, a PCP’s diagnosis of SLE appeared to significantly vary by the race and sex of the patient in the vignette. The highest accuracy at initial diagnosis occurred for Black female cases and persisted at the final diagnosis even after reviewing additional lab results. The lowest accuracy at initial diagnosis occurred for White male cases. Further, review times for correct initial and final diagnoses varied by race-sex version, with the longest times for White male cases. Assessment of planned next steps indicated participants who listed SLE as a differential diagnosis were more likely to check ANA levels for White male cases. While these findings reflect diagnostic tendencies and cognitive processes under standardized, vignette-based conditions, they nonetheless highlight the need to identify strategies for addressing diagnostic inaccuracies and promoting equitable diagnostic processes for all patients.

As one might expect, we observed that PCPs variable diagnostic accuracy by race and sex aligns with the distribution of SLE’s descriptive epidemiology, despite all other elements of the clinical vignette being held constant. The evidence base that informs clinical training, and thereby development of cognitive heuristics, characterizes SLE as a female-predominant disease disproportionately affecting populations of color [1,2,12]. The greater likelihood of PCPs to underdiagnose SLE in male cases, may suggest that diagnostic disparities in SLE are partly influenced by a conscious or unconscious reliance on the representativeness heuristic. In other words, PCPs may be using a cognitive shortcut that assesses the probability of SLE based on its typical presentation and population-level prevalence, which could impact their diagnostic decisions [13]. By relying, and perhaps over-relying on their prior, they may miss, or undervalue, important clinical details in the male cases, for example. Further, our finding that PCPs required more time to correctly diagnose SLE for White male cases may demonstrate that it takes longer to overcome the heuristics and potential bias of assuming it is a disease mostly affecting women, and predominantly women of color.

Evidently, new information detailing lab results that would be frequently observed in SLE (ANA 1:320) and eliminate some differential diagnoses, did not modify the race and sex distribution of correct SLE diagnoses. It is plausible that PCPs did not sufficiently adjust to the additional information provided in the lab results when determining their final diagnosis (i.e., anchoring and inappropriate adjustment heuristic). In fact, PCPs’ review time at final diagnosis was markedly faster compared to initial diagnosis and showed less variability among race and sex versions of the case. These findings may emphasize a need to develop mitigation strategies, such as decision support tools, that prompt physicians to consider a diverse clinical presentation of SLE. For example, evidence based on simulated cases suggests that physician use of artificial intelligence (AI) for decision support could enhance diagnostic reasoning and accuracy [14–16]. Nonetheless, many questions and concerns remain, including the susceptibility of large language models to existing bias, provision of physician training on AI prompting techniques, and protection of patient confidentiality [14,15]. We should also be cognizant of patients’ reciprocal use of AI which, on one hand, could shorten the time to diagnosis, but could also perpetuate misinformation.

Our examination of PCPs’ next steps after initial case presentation offered insight into their diagnostic certainty. Most PCPs elected to order laboratory tests and ANA levels were requested by 79% of participants with SLE as an initial differential diagnosis; interestingly this proportion was highest for the participants who viewed the White male vignette. In contrast, only half of PCPs ordered ANA levels when SLE was not on their initial differential diagnosis. Taken together, White male cases were the least likely to receive a correct diagnosis, yet when SLE was a differential diagnosis, they were the most likely to have ANA checked. This may represent lower certainty in the diagnosis as respondents may have needed more evidence despite otherwise identical clinical findings. Though as noted, additional lab results did not modify the race and sex distribution of correct SLE diagnoses at the final stage. These findings may also reflect racial differences in the provision of diagnostic tests. A 2024 cross-sectional analysis using US emergency and inpatient claims data reported that Black patients were significantly less likely than White patients to receive related diagnostic tests for non-specific discharge diagnoses [17]. Authors postulated these disparities may be shaped by race-based differences in triage practices, patients’ access to primary care, and implicit bias in clinical decision-making [17]. Future research, including the use of qualitative methods, to understand the causes and consequences of racial disparities in the provision of appropriate diagnostic testing for SLE is needed. This gap in practice may also reflect a broader issue in primary care regarding the recognition of autoimmune diseases. Implementing automated clinical reminders and reinforcing guideline-based care may help mitigate these discrepancies.

Delays in SLE diagnosis within primary care may contribute to health disparities, particularly for less representative cases. A cross-sectional study estimated the median time to SLE diagnosis after symptom onset was four years and longer delays were associated with worse clinical outcomes [18]. Diagnostic delays in SLE can be attributed to the absence of a definitive diagnostic test and sometimes non-specific initial symptoms. Our factorial survey experiment suggests there may also be a role for cognitive errors during clinical decision-making. In our study, only 60% of male cases received a final diagnosis of SLE and overall, very few cases were referred to rheumatology (8%). If SLE goes undiagnosed and untreated, it may worsen over time and evolve into more severe phenotypes [19,20]. Thus, if the diagnostic threshold for males is higher, this difference could contribute to males experiencing more severe phenotypes and damage at diagnosis, ultimately leading to poorer prognosis. Indeed, an analysis of a population-based lupus registry showed that males had a 1.6-fold higher rate of lupus nephritis and a 2.1-fold increased rate of thrombocytopenia compared to females [3]. Nevertheless, males with SLE may also have an intrinsically more severe phenotype independent of cognitive bias.

A major strength of this research is the study design, which jointly randomized the “exposure” of race (Black/White) and sex (male/female) while holding constant all clinical context of the case vignette. We incorporated a secondary measure of review time that is consistent with other measures of implicit bias but also considers cognitive load [21]. Cognitive heuristics often reduce cognitive load; however, our measure of review time may demonstrate that the effort required to override cognitive heuristics might increase a physicians’ cognitive load. There are also limitations to consider. First, case vignettes do not fully represent real-world clinical practice and rather reflect diagnostic tendencies and cognitive processes elicited under controlled vignette-based conditions. The clinical vignettes and the format may trigger exam-based thinking in participants, inadvertently leading them to rely more on cognitive shortcuts and descriptive epidemiology. We also did not consider how physician personality traits influence medical decisions. A systematic review found low tolerance to risk and overconfidence was associated with diagnostic inaccuracies [22]. Regardless, our randomized design is presumed to have equally distributed these personality traits and other confounding variables across race-sex case versions. Although respondents comprised a small percentage of contacted PCPs, our sample was generally representative of the internal and family medicine workforce per the 2023 American Association of Medical Colleges (AAMC) workforce dashboard [23]. The AAMC dashboard indicates a higher proportion of male PCPs (60.1% internal medicine, 56.2% family medicine) and a majority aged 40–64 years (57.5% internal medicine, 58.6% family medicine), while our sample had 57.0% men with an average age of 52 years. Racial distribution was somewhat similar, with 67.9% white, 24.4% Asian, and 2.4% Black in our sample, compared to 57.9% white, 15.3% Asian, and 6.2% Black in the US family medicine workforce (10–14% of AAMC race and ethnicity data unknown) [23].

Our factorial survey experiment demonstrates that a case vignette’s race and sex significantly influence a PCP’s likelihood of correctly diagnosing SLE despite vignettes having otherwise identical clinical information. In practice, this may have negative consequences for patient care and clinical prognosis. Thus, efforts to reduce known health disparities among the SLE population should consider the role of cognitive biases in clinical decision-making. In acknowledging both the difficulties in diagnosing SLE along with the critical role PCPs occupy in facilitating early diagnosis and treatment, it is essential to explore opportunities that improve the diagnosis of SLE in primary care. An emerging opportunity, the integration of AI into physician diagnostic decision-making, requires thoughtful consideration of how to best implement it in clinical practice, ensuring that it enhances patient care by balancing accuracy, usability, and ethical concerns.

## Supporting information

S1 Appendix Systemic lupus erythematosus case description. (DOCX)

S2 Appendix Classification of participant responses. (DOCX)

S1 Fig Overview of factorial survey experiment. Abbreviations: ANA – antinuclear antibody; CRP – C-reactive protein; ESR – erythrocyte sedimentation rate; PCP – primary care physician; SLE – systemic lupus erythematosus.
(TIF)

S2 Fig Flow of primary care physician (PCP) survey email invitations, survey completion, and eligibility. (TIF)

S1 Table Participant demographics stratified according to randomly assigned case vignette (n = 1031). (DOCX)

S2 Table Participants (n = 1031) initial diagnoses listed in their overall differential, independent of rank, presented according to jointly randomized race and sex version. (DOCX)
