# Supplementary material for: Effect of race and sex on lupus diagnosis in primary care: A randomized factorial survey study
Source: PLoS One. 2026 Feb 6;21(2):e0342328. doi: 10.1371/journal.pone.0342328 (PMC12880670; doi:10.1371/journal.pone.0342328)
Supplement: S1 Appendix — (DOCX) [file pone.0342328.s001.docx]

**S1 Appendix. Systemic lupus erythematosus case description**

Part 1

A 32 year-old [INSERT RACE] [INSERT SEX] is evaluated in the urgent care clinic for a 1 week history of fever, joint pain, and fatigue. She/He complains of several months of decreased energy, as well as intermittent joint pain with puffiness in her/his hands and feet. For the past week, she/he has been experiencing stiffness in his hands and feet for 30-45 minutes, worse in the morning. He endorses photosensitivity but no rash. She/He also mentions a few “canker sores” over the past few months. Her/His past medical history is notable only for acne as a teenager. She/He does not drink, smoke, or use illicit drugs. She/He lives with a roommate who had a recent brief upper respiratory syndrome but otherwise no sick contacts. Her/His father has rheumatoid arthritis. Remaining family history is negative. She/He takes no medications.

Physical exam: BMI 29
Vitals at rest/sitting: T 38C (100.4F), BP 125/70, HR 95, RR 16
General: not in acute distress, appears at the stated age
HEENT: No conjunctival pallor, cervical lymphadenopathy or thyromegaly. There is a shallow-based ulcer along the right buccal mucosa.

Lungs: clear to auscultation bilaterally. No crackles.
Cardiac: regular rhythm. S1/S2 appreciated in all fields. Normal pulses.
Neuro: cranial nerves 2-12 intact, no gross motor deficits

MSK: tenderness to palpation along several metacarpophalangeal and proximal interphalangeal joints bilaterally, several with mild synovitis. No joint deformities. No peripheral edema
Skin: no rash
Psych: normal affect, linear and non-tangential thoughts, normal speech

Part 2

The following labs come back a few days after you see this patient.

Laboratory studies:
Leukocyte count 2.9 (4.0-11.0 K/uL)
Hemoglobin 13.0 (13.5-17.7 g/dL)
Platelet count 160 (150-400 K/uL)
Serum creatinine 0.8 mg/dL (0.5-1.2 mg/dL)
ESR 37 (0-31 mm/hr)
CRP 1.6 (<0.9mg/dL)
ANA 1:320 in homogeneous pattern
Rheumatoid factor negative
CCP antibodies negative
HIV-1/2 antibodies negative
Parvovirus B19 IgG, IgM pending EBV IgG and IgM pending
