# Supplementary material for: Effect of race and sex on lupus diagnosis in primary care: A randomized factorial survey study
Source: PLoS One. 2026 Feb 6;21(2):e0342328. doi: 10.1371/journal.pone.0342328 (PMC12880670; doi:10.1371/journal.pone.0342328)
Supplement: S2 Appendix — (DOCX) [file pone.0342328.s002.docx]

**S2 Appendix: Classification of participant responses**

**Initial diagnoses.** We began by identifying participants who entered ***SLE*** (e.g., “lupus”, “SLE”, “systemic lupus erythematosus”) as a correct 1^st^ diagnosis, 2^nd^ diagnosis, or 3^rd^ diagnosis. Misspelled responses suggestive of SLE (e.g., “lupas”, “SLA”, “les”) were reviewed with co-authors (AH, JS) and the consensus was to classify such entries as correct. If a participant repeated the diagnosis of SLE in all three diagnostic rankings, SLE was considered the participants’ first choice and the repetition of SLE in the 2^nd^ and 3^rd^ positions were dismissed. In addition, we created a binary variable to indicate whether participants included SLE in their top three differential irrespective of ranking.

We then used an inductive approach to classify incorrect initial diagnoses as binary indicator variables. Several participants entered ***other rheumatic diseases****,* which included “rheumatoid arthritis, “Bechet’s”, “vasculitis”, “connective tissue disease”, among many others. ***Infectious arthritis*** was another common incorrect diagnosis, with entries such as “reactive arthritis”, “viral associated arthritis”, “infective arthritis”, and “viral induced arthropathy” were included in this category. This category was differentiated from any general infection that was viral or bacterial in origin that did not include mention of arthritis, arthropathy, or synovitis. Rather, entries classified as ***general infections*** included for example “parvovirus”, “viral syndrome”, “influenza”, “EBV”, and “endocarditis”. ***Sexually transmitted infections*** constituted its own category separate from general infections, and included “HIV”, “syphilis”, “gonorrhea”, “HSV”, “chlamydia”, and “STD/STI”. A number of participants mentioned incorrect differential diagnoses that were ***endocrine disorders***, the most common being “hypothyroidism”, along with mentions of “diabetes”, hormonal changes”, “pituitary issues”, and general “endocri[ne] deficiencies”. Some incorrect responses generally stated ***autoimmune disease*** (e.g., “autoimmune”, “autoimmune disease”, “autoimmune non-specific”). We also formed a category to specifically capture responses that described ***autoimmune or autoinflammatory gastrointestinal disorders***, namely “inflammatory bowel disease”, “Crohn’s”, “ulcerative colitis”, and “celiac disease”. Additional categories to classify incorrect responses were ***Lyme disease*** (e.g., “lyme”, “tick borne illness”), ***sleep apnea*** (e.g., “OSA”, “sleep apnea”), ***sarcoidosis***, and ***mental health*** (e.g., “depression”, “anxiety”, “stress”). Finally, we established a ***miscellaneous*** category for responses that were independent, infrequent, or limited to describing symptoms (e.g., “cancer”, “renal failure”, “anemia”, “over weight”, “vitamin deficiency”, “allergies”, “joint pain”, “myalgias”)

**Final diagnosis.** We first reviewed entries for correct final diagnoses of SLE, either singularly or as a differential diagnosis, and labelled these correct responses as ***SLE***. Correct responses included “lupus”, “SLE”, “lupus arthritis”, and “systemic lupus erythematosus”. Misspelled responses suggestive of SLE (e.g., “lupas”) were reviewed with co-authors (AH, JS) and the consensus was to classify such entries as correct.

Once all correct diagnoses were identified and coding was reviewed, we applied an inductive approach to classify the incorrect responses. The most common incorrect diagnoses were ***other rheumatic disease***, which captured responses such as “rheumatoid arthritis”, “connective tissue disease”, “Sjogren’s”, and “Bechet’s”. Several participants had entries of “reactive arthritis”, “viral associated polyarthritis”, or “infectious related polyarthritis”, which we collectively grouped as ***infectious arthritis***. Responses pertaining to non-rheumatic ***general infections*** were also common (e.g., “EBV”, “parvovirus”, “viral infection”). A few participants indicated a broad and non-specific diagnosis of ***autoimmune disease*** (e.g., “autoimmune disorder”, “autoimmune dz”). Responses that were infrequent and/or unconnected, such as “kidney ds” and “hematologic malignancy”, were classified under ***miscellaneous***. Finally, some participants to a certain extent indicated they “do not know”, and these responses were classified as ***unknown***.

The classification of all correct and incorrect responses enabled us to then identify participants who correctly and singularly identified SLE as the final diagnosis (***SLE-specific****)* and those that may have included SLE along with a differential diagnosis (***SLE (+/-differential)****).*

**Next steps (initiate treatment).** First, responses from participants indicating that their next step would involve initiating treatment were reviewed for appropriateness. One entry that instead described tests to be ordered (“Inflammatory arthritis workup…”) was reassigned to the ‘order tests’ category. We then applied an inductive approach to classifying treatments into binary categorical variables, which were not mutually exclusive given participants could list ≥1 treatment. The most common treatment category was ***NSAIDs*** and included responses such as “NSAID”, “ibuprofen”, “aspirin”, “naproxen”, and “anti-inflammatory”. Participants electing to initiate treatment also often chose to proceed with ***corticosteroids*** (e.g., “prednisone”, “corticosteroids”, “steroids”). Entries such as “Tylenol”, “acetaminophen”, and “antipyretics” were grouped together as ***acetaminophen***. Some participants wrote “hydroxychloroquine”, “Plaquenil”, “methotrexate”, and broadly “DMARDs”, which we then categorized as ***DMARDs***. The category ***Synthroid*** captures participant entries of “levothyroxine”, “Synthroid”, and “thyroid replacement”. We also assigned participant response variables for treatments classified as ***antibiotics*** (e.g., “doxycycline”, “augmentin”, “antibiotics”), ***topical pain relief*** (e.g., “voltaren gel”, “topical lidocaine”, “pain cream), ***oral pain relief*** (e.g., “viscous lidocaine”, “triamcinolone in Orabase”), and lastly ***antivirals*** (e.g., “acyclovir”).

**Next steps (send referral).** We reviewed text entries for participants who indicated that one or more of their next steps included sending a referral. The most common referral was for ***rheumatology*** (e.g., “rheum”, “rheumatologist”, “rheumatology”). The remaining uncategorized referrals were to “ophthalmology”, “infectious disease”, “orthopedics”, “PT”, “periodontist”, and “dentist”. Some participants entered more than one referral (e.g., “Rheumatology. Orthopedics”) and therefore these binary variables are not mutually exclusive.

**Next steps (order tests).** Most participants ordered tests, which included lab tests and/or imaging. Along with evaluating specific entries for tests ordered, we also scanned participant entries under referrals and treatments given some participants included tests to be ordered under these headings. We established a binary variable to describe whether participants ordered an ***ANA*** test, which captured responses of “ANA”, “antinuclear antibody”, and “ANA panel”. Further, there were a number of entries that referenced some variation of a ***rheumatology panel***, which for instance included “arthritis panel”, “rheumatology panel”, “autoimmune labs”, and “lupus panel”.
