# Supplementary figures and images for: Effect of race and sex on lupus diagnosis in primary care: A randomized factorial survey study
Source: PLoS One. 2026 Feb 6;21(2):e0342328. doi: 10.1371/journal.pone.0342328 (PMC12880670; doi:10.1371/journal.pone.0342328)

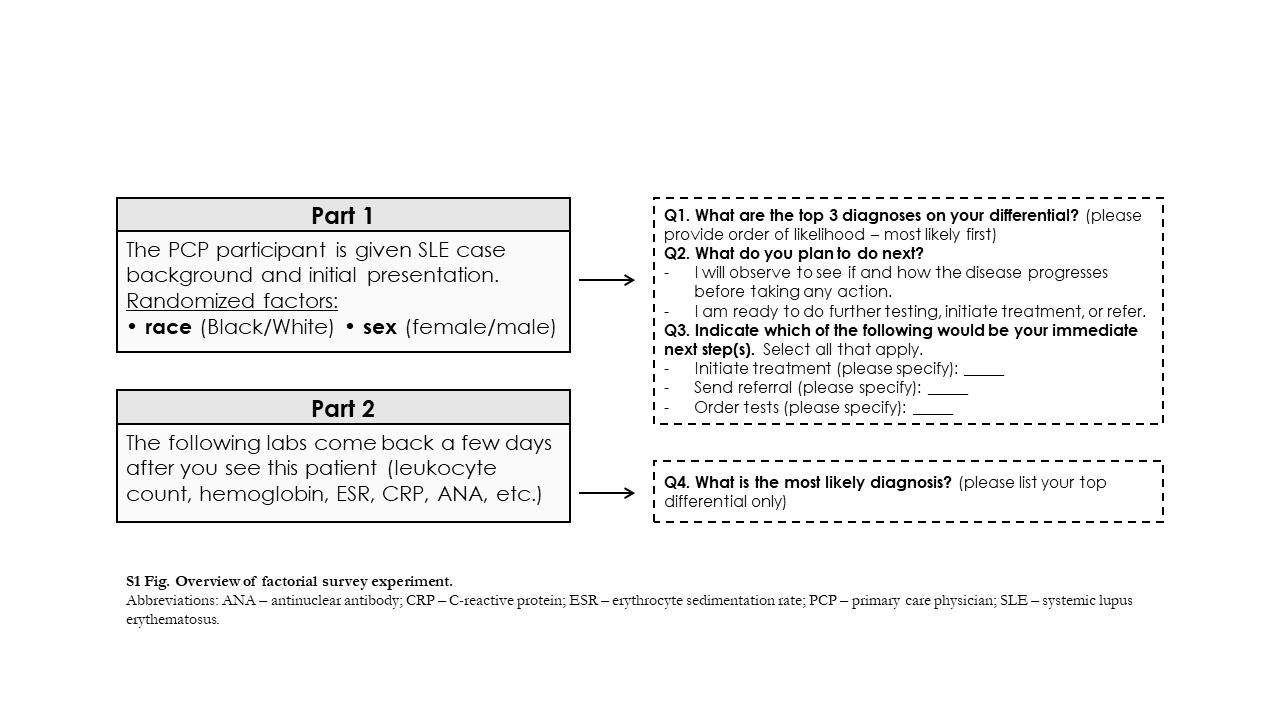

Supplement: S1 Fig — Abbreviations: ANA – antinuclear antibody; CRP – C-reactive protein; ESR – erythrocyte sedimentation rate; PCP – primary care physician; SLE – systemic lupus erythematosus. (TIF) [file pone.0342328.s003.tif]

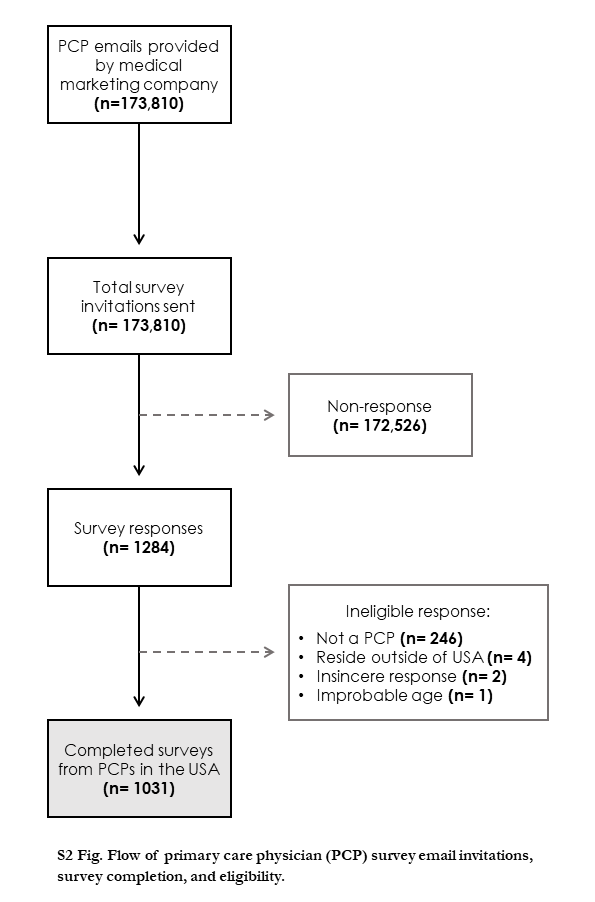

Supplement: S2 Fig — (TIF) [file pone.0342328.s004.tif]
