# Supplementary material for: Effect of race and sex on lupus diagnosis in primary care: A randomized factorial survey study
Source: PLoS One. 2026 Feb 6;21(2):e0342328. doi: 10.1371/journal.pone.0342328 (PMC12880670; doi:10.1371/journal.pone.0342328)
Supplement: S1 Table — (DOCX) [file pone.0342328.s005.docx]

| **S1 Table.** Participant demographics stratified according to randomly assigned case vignette (n=1031) | | | | | |
| --- | --- | --- | --- | --- | --- |
|  | **Characteristic** | **Black female**  **(n=263)** | **White female**  **(n=253)** | **Black**  **male**  **(n=253)** | **White**  **male**  **(n=262)** |
| Age, mean (SD) | | 52.5 (12.6) | 52.3 (12.2) | 51.0 (11.3) | 52.5 (12.2) |
| Gender, No. (%) | |  |  |  |  |
|  | Woman | 110 (41.8) | 116 (45.9) | 112 (44.3) | 102 (38.9) |
|  | Man | 153 (58.2) | 135 (53.3) | 135 (53.4) | 160 (61.1) |
|  | Not listed | 0 (0.0) | 2 (0.8) | 1 (0.4) | 0 (0.0) |
| Race, No. (%) | |  |  |  |  |
|  | White | 188 (71.5) | 179 (70.8) | 160 (63.2) | 173 (66.0) |
|  | Asian | 53 (20.2) | 61 (24.1) | 71 (28.1) | 67 (25.6) |
|  | Black | 7 (2.7) | 5 (2.0) | 4 (1.6) | 9 (3.4) |
|  | American Indian or Alaska Native | 2 (0.8) | 0 (0.0) | 0 (0.0) | 0 (0.0) |
|  | Pacific Islander or Native Hawaiian | 0 (0.0) | 0 (0.0) | 2 (0.8) | 0 (0.0) |
|  | ≥2 selected | 4 (1.5) | 6 (2.4) | 3 (1.2) | 4 (1.5) |
|  | Not listed | 9 (3.4) | 2 (0.8) | 13 (5.1) | 9 (3.4) |
| Ethnicity, No. (%) | |  |  |  |  |
|  | Hispanic | 10 (3.8) | 14 (5.5) | 11 (4.4) | 15 (5.7) |
|  | Not Hispanic | 253 (96.2) | 239 (94.5) | 242 (95.7) | 247 (94.3) |
| Medical school, No. (%) | |  |  |  |  |
|  | US | 203 (77.2) | 185 (73.1) | 186 (73.5) | 190 (72.5) |
|  | International | 60 (22.8) | 68 (26.9) | 67 (26.5) | 72 (27.5) |
| Years since residency, No. (%) | |  |  |  |  |
|  | Currently in residency | 6 (2.3) | 5 (2.0) | 6 (2.4) | 11 (4.2) |
|  | <5 years | 18 (6.8) | 24 (9.5) | 21 (8.3) | 17 (6.5) |
|  | 5-10 years | 41 (15.6) | 29 (11.5) | 33 (13.0) | 32 (12.2) |
|  | 11-20 years | 72 (27.4) | 74 (29.3) | 80 (31.6) | 72 (27.5) |
|  | >20 years | 126 (47.9) | 121 (47.8) | 113 (44.7) | 130 (49.6) |
| Practice setting, No. (%) | |  |  |  |  |
|  | Private group practice | 92 (35.0) | 93 (36.8) | 97 (38.3) | 91 (34.7) |
|  | Academic | 75 (28.5) | 84 (33.2) | 79 (31.2) | 79 (30.2) |
|  | Multiple settings | 28 (10.7) | 29 (11.5) | 33 (13.0) | 35 (13.4) |
|  | City or County public hospital | 25 (9.5) | 23 (9.1) | 18 (7.1) | 19 (7.3) |
|  | Retired | 16 (6.1) | 9 (3.7) | 7 (2.8) | 10 (3.8) |
|  | Veteran Affairs | 13 (4.9) | 6 (2.4) | 9 (3.6) | 10 (3.8) |
|  | HMO | 11 (4.2) | 4 (1.6) | 7 (2.8) | 10 (3.8) |
|  | Military | 0 (0.0) | 1 (0.4) | 0 (0.0) | 0 (0.0) |
|  | Not working (looking to work) | 0 (0.0) | 1 (0.4) | 0 (0.0) | 3 (1.2) |
|  | Not working | 0 (0.0) | 0 (0.0) | 1 (0.4) | 3 (1.2) |
|  | Not clinically practicing | 3 (1.1) | 3 (1.2) | 2 (0.8) | 2 (0.8) |
| Clinical work, No. (%) | |  |  |  |  |
|  | Patient care | 224 (85.2) | 218 (86.2) | 220 (87.0) | 221 (84.4) |
|  | Administrative work | 17 (6.5) | 14 (5.5) | 11 (4.4) | 20 (7.6) |
|  | Education | 14 (5.3) | 14 (5.5) | 17 (6.7) | 15 (5.7) |
|  | Research | 8 (3.0) | 7 (2.8) | 5 (2.0) | 6 (2.3) |
| Location of current practice, No. (%) | |  |  |  |  |
|  | Urban | 104 (39.5) | 114 (45.1) | 115 (45.5) | 125 (47.7) |
|  | Suburban | 117 (44.5) | 100 (39.5) | 100 (39.5) | 92 (35.1) |
|  | Rural | 42 (16.0) | 39 (15.4) | 38 (15.0) | 45 (17.2) |
| US geographic division, No. (%) | |  |  |  |  |
|  | South Atlantic | 45 (17.1) | 46 (18.2) | 47 (18.6) | 44 (16.8) |
|  | Pacific | 43 (16.4) | 38 (15.0) | 59 (23.3) | 39 (14.9) |
|  | Middle Atlantic | 39 (14.8) | 37 (14.6) | 29 (11.5) | 42 (16.0) |
|  | East North Central | 32 (14.8) | 33 (13.0) | 35 (13.8) | 38 (14.5) |
|  | West North Central | 38 (14.5) | 31 (12.3) | 22 (8.7) | 32 (12.2) |
|  | Mountain | 25 (9.5) | 28 (11.1) | 19 (7.5) | 22 (8.4) |
|  | West South Central | 13 (4.9) | 18 (7.1) | 19 (7.5) | 21 (8.0) |
|  | New England | 21 (8.0) | 11 (4.4) | 15 (5.9) | 17 (6.5) |
|  | East South Central | 7 (2.7) | 11 (4.4) | 8 (3.4) | 7 (2.7) |
|  | Puerto Rico | 0 (0.0) | 0 (0.0) | 0 (0.0) | 0 (0.0) |
| Abbreviations: SD – standard deviation | | |  |  |  |
