# Supplementary material for: Effect of race and sex on lupus diagnosis in primary care: A randomized factorial survey study
Source: PLoS One. 2026 Feb 6;21(2):e0342328. doi: 10.1371/journal.pone.0342328 (PMC12880670; doi:10.1371/journal.pone.0342328)
Supplement: S2 Table — (DOCX) [file pone.0342328.s006.docx]

| **S2 Table.** Participants (n=1031) initial diagnoses listed in their overall differential, independent of rank, presented according to jointly randomized race and sex version. | | | | |
| --- | --- | --- | --- | --- |
|  | **Black Female**  **(n=263)** | **White Female**  **(n=253)** | **Black**  **Male**  **(n=253)** | **White**  **Male**  **(n=262)** |
| **Initial differential diagnoses (overall), n (%)** | | | | |
| SLE | 190 (72.2) | 172 (68.0) | 152 (60.1) | 145 (55.3) |
| Other rheumatic disease | 240 (91.3) | 233 (92.1) | 230 (90.9) | 248 (94.7) |
| Infectious arthritis | 57 (21.7) | 46 (18.2) | 75 (29.6) | 69 (26.3) |
| General infection^a^ | 48 (18.3) | 64 (25.3) | 51 (20.2) | 64 (24.4) |
| Sexually transmitted infection | 23 (8.8) | 13 (5.1) | 40 (15.8) | 31 (11.8) |
| General autoimmune disease | 21 (8.0) | 15 (5.9) | 9 (3.6) | 20 (7.6) |
| Gastrointestinal autoimmune | 4 (1.5) | 7 (2.8) | 8 (3.2) | 9 (3.4) |
| Endocrine disorder | 15 (5.7) | 17 (6.7) | 8 (3.2) | 8 (3.1) |
| Lyme disease | 22 (8.4) | 15 (5.9) | 19 (7.5) | 21 (8.0) |
| Sleep apnea | 2 (0.8) | 5 (2.0) | 3 (1.2) | 0 (0.0) |
| Sarcoidosis | 10 (3.8) | 1 (0.4) | 9 (3.6) | 1 (0.4) |
| Mental health | 4 (1.5) | 3 (1.2) | 3 (1.2) | 0 (0.0) |
| Miscellaneous | 23 (8.8) | 24 (9.5) | 22 (8.7) | 24 (9.2) |
| *^a^General infections could be viral or bacterial in origin.* | | | | |
